# Expression Profiling and Proteomic Analysis of JIN Chinese Herbal Formula in Lung Carcinoma H460 Xenografts

**DOI:** 10.1155/2013/160168

**Published:** 2013-08-20

**Authors:** Luyu Zheng, Weiyi Zhang, Miao Jiang, Huarong Zhang, Fei Xiong, Yang Yu, Meijuan Chen, Jing Zhou, Xiaoming Dai, Yuping Tang, Ming Jiang, Mingyan Wang, Ge Cheng, Jinao Duan, Wei Yu, Biaoyang Lin, Haian Fu, Xu Zhang

**Affiliations:** ^1^College of Basic Medicine and School of Pharmacy, Nanjing University of Chinese Medicine, Nanjing 210029, China; ^2^Zhejiang California International Nanosystems Institute, Zhejiang University, Hangzhou 310058, China; ^3^School of Chinese Medicine, Hong Kong Baptist University, Hong Kong; ^4^Department of Pharmacology and Winship Cancer Institute, Emory University School of Medicine, 1510 Clifton Road, Atlanta, GA 30322, USA

## Abstract

Many traditional Chinese medicine (TCM) formulae have been used in cancer therapy. The JIN formula is an ancient herbal formula recorded in the classic TCM book *Jin Kui Yao Lue* (Golden Chamber). The JIN formula significantly delayed the growth of subcutaneous human H460 xenografted tumors in vivo compared with the growth of mock controls. Gene array analysis of signal transduction in cancer showed that the JIN formula acted on multiple targets such as the mitogen-activated protein kinase, hedgehog, and Wnt signaling pathways. The coformula treatment of JIN and diamminedichloroplatinum (DDP) affected the stress/heat shock pathway. Proteomic analysis showed 36 and 84 differentially expressed proteins between the mock and DDP groups and between the mock and JIN groups, respectively. GoMiner analysis revealed that the differentially expressed proteins between the JIN and mock groups were enriched during cellular metabolic processes, and so forth. The ones between the DDP and mock groups were enriched during protein-DNA complex assembly, and so forth. Most downregulated proteins in the JIN group were heat shock proteins (HSPs) such as HSP90AA1 and HSPA1B, which could be used as markers to monitor responses to the JIN formula therapy. The mechanism of action of the JIN formula on HSP proteins warrants further investigation.

## 1. Introduction

Nonsmall cell lung cancer is a major cause of cancer-related mortality worldwide and is highly resistant to treatment by classical cytotoxic agents including platinum-based drugs. Traditional Chinese medicine (TCM) aims to correct maladjustments and restore the self-regulatory ability of the body without antagonizing specific pathogenic targets. TCM has been used to treat human diseases and has a long history of safety and efficacy. The approaches used in systems biology and pharmacogenetics are similar to the practices of TCM [1]. The JIN formula is an ancient herbal formula recorded in the classical TCM book *Jin Kui Yao Lue* (Golden Chamber). This formula is composed of *Ophiopogon japonicus* (30 g), prepared *Rhizoma pinelliae* (15 g), *Ginseng radix* (30 g), *Glycyrrhiza radix* (12 g), peach kernel (15 g), unprepared *Coix lacryma-jobi* seeds (30 g), Chinese wax gourd seed (30 g), and *Phragmititis caulis* (30 g). TCM theory indicates that lung cancer is related to Qi and Yin deficiencies, Qi insufficiency in the spleen and lungs, or pathological changes because of Qi stagnation, blood stasis, and phlegm and toxin accumulation. The JIN formula can replenish both Qi and Yin, strengthen the spleen and lungs, and clear the lungs. The JIN formula can also remove phlegm and activate blood circulation to remove stasis. This study focuses on the efficacy of the JIN formula in vivo in a murine xenograft lung cancer model. This study explains the underlying mechanism of the JIN formula by microarray and proteomic analysis.

## 2. Materials and Methods

### 2.1. Cell Line and Culture Conditions

Human lung carcinoma (NCI-H460) cells were obtained from the Cell Line Bank (Shanghai, China) and used in the described experiments. The cell line was cultured in Dulbecco's modified Eagle's medium (Gibco, USA) with 10% fetal bovine serum, 100 U/mL penicillin, and 100 mg/mL streptomycin in a cell culture incubator at 37°C under 5% CO_2._ The cells were used within 2 passages to 4 passages at the log phase of growth. Aliquots of the cell line were frozen at −80°C until use.

### 2.2. Experimental Animals

Balb/c athymic (nude) mice (male, 6 wk to 8 wk, *n* = 30) weighing 21 g to 25 g were purchased from the Animal Center of the Academy of Military Medical Sciences (Beijing, China). The mice were housed under specific pathogen-free conditions according to the guidelines of the Institutional Animal Care and Use Committee of Nanjing University of Chinese Medicine. The animal room was controlled for temperature (22 ± 2°C), light (12 h light/dark cycle), and humidity (50 ± 10%). All laboratory feed pellets and bedding were autoclaved.

### 2.3. Xenograft Model

The tumor regression model was successfully applied in nude mice to evaluate antitumor activity. This model was used to evaluate the suppression of solid tumor growth by the JIN formula. A total of 1 × 10^7^ NCI-H460 cells in 0.2 mL of culture medium were injected subcutaneously into the flank of each mouse by using a 26 ga needle. After 7 d of observation, a solid tumor mass was excised from the mice inoculated with NCI-H460 cells. When the tumor volume in the nude mice reached approximately 50 mm^3^, the xenografted tumor models were randomly distributed into four groups: NCI-H460 + saline (mock group), NCI-H460 + 12 mg/mL/d JIN formula (JIN group), NCI-H460 + 20 *μ*g/mL/d diamminedichloroplatinum (DDP) (DDP group), NCI-H460 + JIN formula + DDP (coformulated group, same dosage as in other groups), and saline (control group, without tumor). Six mice were included in each group. The JIN formula was orally administered daily for 15 d, and DDP was injected abdominally for 15 d. A group with no tumor was administrated orally with saline and served as a control group.

### 2.4. Antitumor Activity In Vivo

All animals were monitored for activity, physical condition, body weight, and tumor growth. The body weight of each animal was measured once every 3 d. The longest (*A*) and shortest (*B*) tumor diameters (mm) were obtained, and the formula for an ellipsoid sphere (0.52 × *A* × *B* ^2^) was used to calculate the tumor volume every 2 d. The tumor weights were also measured on the basis of the tumor regression on the final day of the experiment after the animals were sacrificed. The antitumor activities of the treatments were expressed as follows: inhibition rate (%) = (*W*_mock_ − *W*_treatment_)/*W*_model_ × 100%, where *W*_mock_ and *W*_treatment_ are the tumor weights of the mock and treatment groups, respectively. The xenografted tumors and other vital organs of the mice were harvested, fixed in 4% formalin, embedded in paraffin, and cut in 4 mm sections for histological study.

### 2.5. Preparation of Total RNA

Total RNA from the xenografted tumor was isolated with TRIZOL reagent (Invitrogen Life Technologies). We pooled an equal amount of cancer tissues from six individual mouse xenografts in each group (JIN, DPP, JIN + DDP, and the mock groups) to save cost. The RNA was eluted in RNase-free water, and the integrity was verified by electrophoresis on 1.2% agarose gel and visualized with ethidium bromide staining. The concentration was quantified by ultraviolet absorption with a nanodrop spectrophotometer (BioLab Ltd.).

### 2.6. Expression Profiling Array

The total RNA from the experimental samples was used as a template and reverse-transcribed to generate cDNA. The cDNA was then converted into biotin-labeled cRNA probes by using Biotin-16-dUTP (Roche) by in vitro transcription with the TrueLabeling-AMP linear RNA amplification kit (SuperArray Bioscience). Before hybridization, the cRNA probes were purified with the ArrayGrade cRNA Cleanup Kit (Superarray Bioscience).

The Oligo GEArray microarray series (OHS-044) (SuperArray Inc.) was used to quantify the expression of the 113 genes involved in the 15 signal transduction pathways in cancer (mitogen-activated protein kinase (MAPK), Wnt, hedgehog, signal transducers, and activators of transcription, stress/heat shock, inflammation/nuclear factor-kappa B, survival, androgen/estrogen, and transforming growth factor-beta (TGF-*β*) pathways, etc).

Purified cRNA probes were hybridized with the membranes at 60°C overnight with slow agitation in a hybridization oven. The hybridized membranes were washed once in saline sodium citrate buffer solution I (2x, 1% sodium citrate/sorbitol buffer) and once in solution II (0.1x, 0.5% sodium dodecyl sulfate (SDS)). Membranes were incubated with alkaline phosphatase-conjugated streptavidin. Thereafter, the membranes were washed and incubated with the CDP-Star chemiluminescent substrate. Detection was performed by exposure to X-ray film. Membrane images were analyzed by the web-based GEArray Expression Analysis Suite software. The relative expression level of each gene was determined by comparing the signal intensity of each gene in the array after background and normalization corrections. For comparison, at least 1 spot intensity had to be more than twice the background intensity, and the spot intensity ratios had to be higher than 2 (for upregulation) or lower than 0.5 and higher than 0 (for downregulation).

### 2.7. Quantitative Real-Time Polymerase Chain Reaction (PCR)

PCR primers were designed by using the Primer 5.0 software (Primer, Canada) based on the special design criteria for real-time PCR primers (Table 1). The forward and reverse primer sequences and lengths of amplified gene products were as follows. The RNA samples were reverse-transcribed by using Moloney murine leukemia virus reverse transcriptase (Epicentre). An oligo-dT primer was used to prime the reverse transcription. Beta-actin was selected as the reference gene for the normalization of results. Quantitative real-time PCR was performed by using Rotor-Gene 3000 real-time PCR (Corbett Research) with SYBR green (Molecular Probes) as the detection system. The results were analyzed by Rotor-Gene 6.0 software (Corbett Research). Relative expression levels were determined in three repeat experiments (*n* = 3, mean ± standard deviation).

### 2.8. Sample Preparation for Proteomic Analysis

For the proteomic analysis, we pooled an equal amount of cancer tissues from six individual mouse xenografts in each group (JIN, DPP, and the mock groups) to save cost. Cancer tissues were snap-frozen in liquid nitrogen and stored in a −80°C freezer. To extract proteins, the cancer tissues were grinded under liquid nitrogen with a mortar and pestle, and tissue lysis buffer was added. The solution was then sonicated on ice with an ultrasonic processor (Bioblock Scientific, France) for 90 min. The tissue lysis buffer contained 50 mM Tris (pH 7.4), 150 mM NaCl, 1% Triton X-100, 1% sodium deoxycholate, 0.1% SDS, 1x cocktail (0.5 mM Na_3_VO_4_, 50 mM NaF, 1x phosphatase inhibitor cocktail, and 5 mM phenylmethanesulfonyl fluoride). The homogenate was then centrifuged at 12,000 g for 1 h at 4°C. Supernatant was collected, and protein concentration was determined by bicinchoninic acid assay. Each sample (70 *μ*g) was separated on 12% SDS polyacrylamide gel. Gels were stained with colloidal coomassie brilliant blue. Twenty-seven individual bands were removed and subjected to gel trypsin digestion by using the In-Gel Tryptic Digestion Kit protocol (Pierce Biotechnology, Rockford, IL, USA). The bands were then analyzed by tandem mass spectrometry (MS/MS).

### 2.9. Mass Spectrometric Analysis

Tryptic peptide mixtures were separated by Ettan multidimensional liquid chromatography (LC) nanoflow/capillary LC system (GE Healthcare, Pittsburgh, PA, USA) equipped with a trapping column (Dionex/LC Packings *μ*-Precolumn Cartridge P/N 160454 C18 PepMap 100; 5 *μ*m, 100 Å, 300 *μ*m internal diameter × 5 mm; Sunnyvale, CA, USA) and nanocolumn (Dionex/LC Packings P/N 160321; 150 mm × 0.075 mm inner diameter, C18 PepMap, 3 *μ*m, 100 Å; Sunnyvale, CA, USA). The mixtures were then analyzed by using LTQ-Orbitrap (Thermo Finnigan, Bremen, Germany) with a nanospray configuration. The precursor ion scan mass spectra (m/z 300–1600) were obtained in the orbitrap with a resolution of *R* = 60,000 at m/z 400, and the number of accumulated ions was 1 × 10^6^. The five most intense ions were isolated and fragmented in a linear ion trap (number of accumulated ions: 3 × 10^4^). The resulting fragment ions were recorded with a resolution of *R* = 15,000 at m/z 400.

### 2.10. Mass Spectra Analysis

The Extract-MSn of BioWorks V3.2 (Thermo Electron, Inc., Waltham, MA, USA) was used to generate the mass spectrometry (MS) peak list with the default parameters. The Interactive Chemical Information System peak detection algorithm was used. The SEQUEST algorithm (Thermo Fisher Inc.) was used for the SEQUEST database search, and the spectra were searched against the IPI.HUMAN.v3.58.fasta protein database (79,794 entries) (http://www.ebi.ac.uk/) by using the BioWorks program V3.2 (Thermo Electron, Inc., Waltham, MA, USA). In the Turbo SEQUEST search parameter setting, the threshold for data generation was 10,000, and the precursor mass tolerance for data generation was 1.4 Da. For the SEQUEST search, peptide tolerance was set at 3 Da, and fragment ion tolerance was set at 0.01 Da. PeptideProphet [2] was used to assess the tandem mass spectra quality. The threshold score for accepting individual tandem mass spectra was *P* = 0.9, which corresponds to a 0.5% error rate in our dataset. One missed tryptic cleavage was permitted.

Carboxyamidomethyl cysteine (Cys_CAM) (+57) was included as a fixed modification for iodoacetamide reduction and alkylation. Given that the proteins were prepared by polyacrylamide gel electrophoresis, cysteines might react with free acrylamide monomers to form propionamide cysteine (Cys_PAM). We included an optional 14 Da in the search to account for potential Cys_PAM. The mass difference between Cys_PAM and Cys_CAM is 14. Methionine oxidation (+15.999 Da) was selected as another optional modification for the database search. Proteins with ProteinProphet *P* value greater than 0.9 and more than two unique peptide hits were considered as true hits. A randomized database of the IPI.HUMAN.v3.58.fasta was used as a decoy database to calculate the false discovery rate of protein identification. The Perl script used for randomization was obtained from http://www.matrixscience.com/downloads/decoy.pl.gz. The false discovery rate (0.568%) was calculated by the ratio of the number of matches in the randomized database to the number of matches in the IPI.HUMAN.v3.58.fasta database. Keratins were removed from the protein list because these proteins often represent contaminations from sample handling. 

The MS/MS data from the cancer and control samples were analyzed by the PeptideProphet and ProteinProphet program for statistical validation by using TPP4.31 [3, 4]. To identify the differential protein expression between two experimental groups (e.g., Sample A versus Sample B), we used the spectral counting method with a Bayesian mixture model [5]. Spectral counts are the average of duplicate LC-MS/MS runs and normalized to the total spectra (5000 × total spectra of the protein/sum of all total spectra of the proteins in the dataset). Significantly differentially expressed proteins were identified with a posterior probability of >0.95 by using the Bayesian mixture model, and the spectral count difference was great than 10. GoMiner [6, 7] was used to find statistically represented gene ontology (GO) biological processes (level 3) with log⁡_10_⁡(*P*) < −2 (i.e., *P* < 0.01).

### 2.11. Western Blot Analysis

Cells were lysed in NP-40 buffer (1.0% NP-40, 10 mM hydroxyethyl piperazine ethanesulfonic acid, pH 7.4, 150 mM NaCl, 5 mM NaF, 2 mM Na_3_VO_4_, 5 mM Na_4_P_2_O_7_, 10 g/mL aprotinin, 10 g/mL leupeptin, and 1 mM phenylmethylsulfonylfluoride). Equal volumes of cell lysate were subjected to SDS-polyacrylamide gel electrophoresis (12.5% gel). Proteins were then electrotransferred into a nitrocellulose membrane (GE Water and Process Technologies, USA). The membranes were blocked in a solution of 5% nonfat dry milk in Tris-buffered saline—Tween 20 buffer (20 mM Tris, pH 7.6, 500 mM NaCl, and 0.5% Tween 20) for 30 min followed by incubation with a primary antibody for at least 2 h. The membrane was then washed and treated with horseradish peroxidase-conjugated anti-mouse immunoglobulin or anti-rabbit immunoglobulin as indicated. Immunodetection was performed by using West Pico (Pierce Chemical, Rockford, IL) or West Dura (Pierce Chemical) followed by imaging on an Image Station 2000R (Eastman Kodak, USA).

### 2.12. Statistical Analysis

The data were analyzed by SPSS 19 software and presented as mean ± standard deviation. The significance of the difference between the mean of the mock and treatment groups was analyzed by using one-way ANOVA followed by Dunnett's *t*-test correction and paired *t*-test. Statistical significance was determined at the level of *P* < 0.05 and *P* < 0.01.

## 3. Results

### 3.1. The JIN Formula Has Similar Effects as the Standard DDP Treatment but Is Less Toxic Than DDP

**Figure 1:**
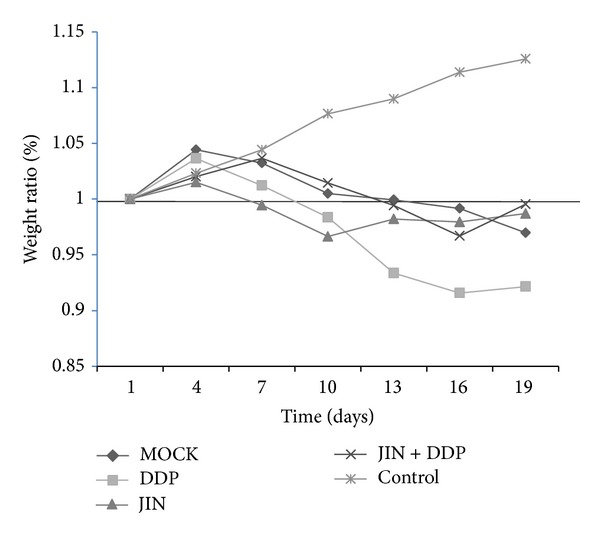
Body weights were measured every 3 days and body weight ratio was calculated relative to baseline measurement. The chemotherapy showed the toxicity to the mice, whereas the herbal treatment showed little toxicity compared to the saline group as measured by body weight loss.

To study the toxicity of the JIN formula, we treated NCI-H460 xenografts that were grown subcutaneously in nude mice with saline, JIN formula, DDP, and JIN formula + DDP (coformulated group) for 15 d. A control group with no tumor was also included. One mouse in the mock group died on the 11th day of treatment. A mouse in the DDP and coformulated group died on the 15th day before sacrifice. Other mice in these two groups significantly lost weight and displayed slower activities and dry skins. All mice in the JIN group were alive and had stable weights, normal activities, and moist skins (Figure 1). 

This result indicated that herbal treatment and chemotherapy prolonged the lifespan of the mice. However, chemotherapy is toxic to the mice, whereas herbal treatment showed little toxicity compared with the saline group as demonstrated by the body weight loss.

**Figure 2:**
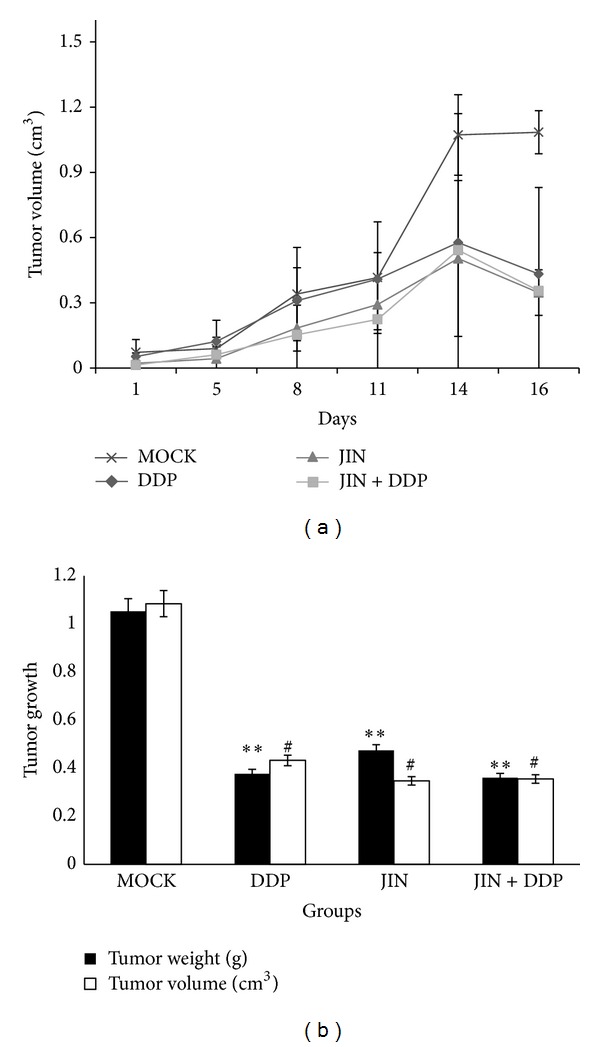
Effect of treatment on tumour growth. (a) The effect of JIN, JIN + DDP, and DDP on tumour size. Tumour volumes were measured every 3 days. (b) The effect of JIN, JIN + DDP, and DDP on tumour weight. Data presented are the mean ± SD at 8–25 days posttumour implantation; groups were compared and analysed using *t*-test. **P < 0.01 tumor weight compared with model group, ^#^ *P* < 0.05 tumor volume compared with model group. DDP, JIN, and coformula inhibited tumor weight by 63.99%, 55.03%, and 65.79%, respectively, (*P* < 0.01) after 15 days of treatment compared to mice administered the vehicle only.

All treatment groups (DDP, JIN, and JIN + DDP) showed inhibited growth of the NCI-H460 cell-transplanted solid tumor compared with the mock group (Figure 2). Significant differences in the final tumor weights (*P* < 0.01) and volume (*P* < 0.01) were found between each treatment group and the mock group after sacrifice (Figure 2). The tumor weights of mice treated with DDP, JIN, and the coformula were inhibited by 63.99%, 55.03%, and 65.79% (*P* < 0.01), respectively, after 15 d of treatment compared with those of mice administered with saline only. 

### 3.2. Histological Changes Induced by JIN Formula Treatment

**Figure 3:**
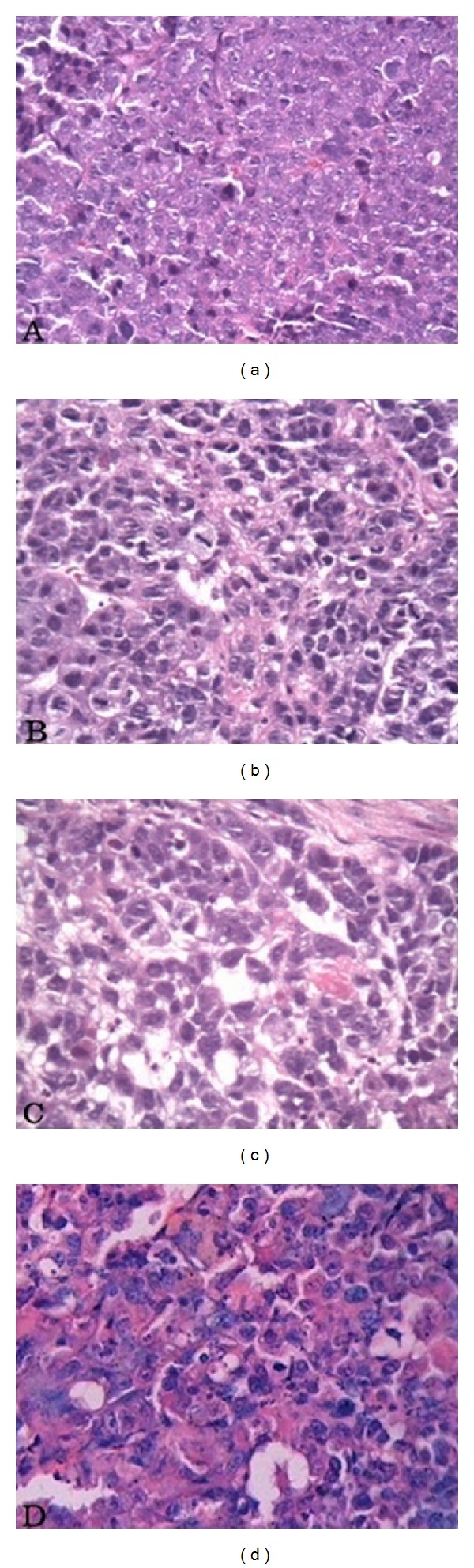
(a) MOCK group; (b) JIN group; (c) DDP group; (d) JIN + DDP group. Histological examination showed the tumors of MOCK group were solid masses composed of densely arranged cells without distinct cell differentiation, which are heteromorphic with a large nucleus containing vesicles and an obvious nucleolus, a reverse proportion between nucleus and cytoplasm, much mitosis and angiogenesis, infiltrative growth of tumor cells, and large areas of necrosis and hemorrhage.

Histological examination showed that the tumors of the mock group were solid masses composed of densely arranged cells without distinct cell differentiations. The cells were heteromorphic and had large nuclei that contain vesicles and obvious nucleoli. Reverse proportions between the nucleus and cytoplasm, high rates of mitosis and angiogenesis, infiltrative growths of tumor cells, and large areas of necrosis and hemorrhage were observed. No significant difference in cell morphology was found between each treatment group and the mock group. However, the tumor cells in the treatment group were less densely arranged, with patchy sparse cell arrangements, enlarged intercellular spaces, vacuoles in the cytoplasms, scattered intense stains in nuclei, scattered pyknosis of tumor cells, and varying degrees of degeneration. Tumor cell metastasis was not found in the lungs and liver (data not shown). The results indicated that the formula can promote the degeneration and death of tumor cells and induce apoptosis, thus inhibiting the growth of lung cancer. These data suggested that the JIN formula could be safely administered as a novel therapeutic agent. Optimization of the dose and dosing schedule might yield a higher antitumor efficacy (Figure 3).

### 3.3. Gene Expression

**Figure 4:**
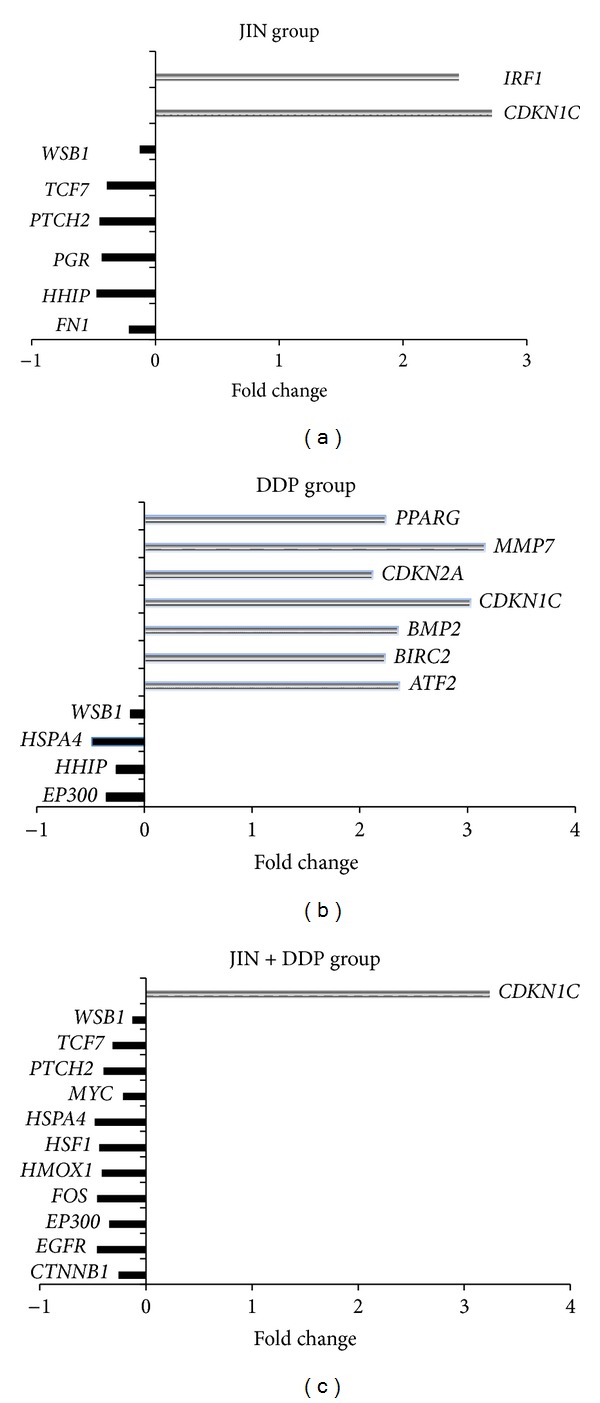
(a) Genes regulated more than 2 folds by Jin treatment on H460 cells. Combined with ratio values 2-fold difference, in JIN group, significantly upregulated gene *CDKN1C* and downregulated genes including *PTCH2*, *TCF7,* and *WSB1* passed statistic *t*-test. The results were reconfirmed by real-time PCR with CDKN1C and PTCH2 primer pairs. (b) Genes regulated more than 2 folds by DDP treatment on H460 cells. (c) The coformula treatment of JIN and DDP also involved the inhibition of the stress/heat shock pathway: *HSPA4*, *HSF1*, *MYC*, *FOS*, and so forth.

To investigate preferentially altered signal transduction pathways in H460 xenografted tumors treated by herbal formula, the Human Q Series Signal Transduction in Cancer Gene Array, which includes marker genes with functions related to cell signal transduction pathways, was used. In the JIN group, the *CDKN1C* gene was upregulated and the *PTCH2*, *TCF7*, and *WSB1* genes were downregulated with twofold differences in ratios, as shown by the *t*-test. The results were reconfirmed by real-time PCR with CDKN1C and PTCH2 primer pairs (Tables 2-3, Figure 4). The expression of these genes indicated the preferential change in the hedgehog and TGF-*β* signaling pathways in H460 xenografted tumor regulated by the JIN formula. 

Furthermore, the coformula treatment of JIN and DDP also inhibited the stress/heat shock pathway, which involves the *HSF1*, *MYC*, and *FOS* genes (Table 4).

### 3.4. Proteomic Analysis of Cancer Cells Treated with JIN and DDP

We conducted a comprehensive mass spectra analysis of the JIN formula, DDP, and mock groups. We identified 1,131, 831, and 1,326 proteins in the JIN formula, DDP, and mock groups, respectively. By using Booth's Bayesian mixture model to compare the spectral count data for shotgun proteomics [5], we identified 40 (corresponding to 36 proteins) and 178 peptides (corresponding to 84 proteins) that were differentially expressed between the mock and DDP groups and between the mock and JIN formula groups, respectively (see Supplementary Tables  1 to 4 available online at http://dx.doi.org/10.1155/2013/160168). We found that the expression of the HSP90AA1 and HSPA1B proteins was significantly reduced in the JIN group compared with that in the mock group. HSPA1B was also significantly reduced in the DDP group compared with that in the mock group. 

GoMiner analysis revealed that the differentially expressed proteins between the JIN and mock groups were enriched in GO:0006977 (DNA damage response signal transduction by p53 class mediator resulting in cell cycle arrest), GO:0006915 (apoptosis), and GO:0016032 (viral reproduction) (Table 5). By contrast, the differentially expressed proteins between the DDP and mock groups were enriched in GO:0065004 (protein-DNA complex assembly) and GO:0006414 (translational elongation) (Table 6). These results suggested that the JIN formula and DPP act on different biological processes. 

**Figure 5:**
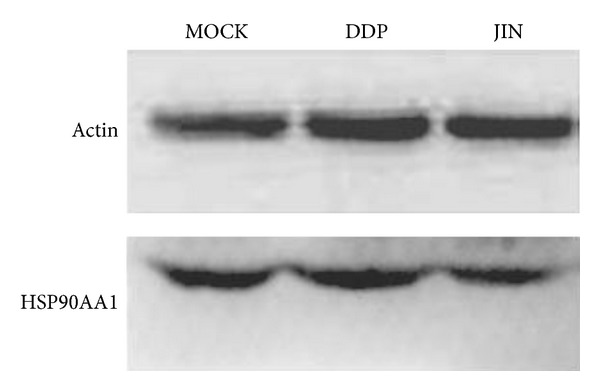
Western blot analysis showing that after treated by JIN formula the HSP90AA1 was downregulated significantly than DDP did.

We identified proteins and protein families that were downregulated in the JIN group compared with these in the mock group. These proteins include several histone family proteins and two heat shock proteins (HSPs), namely, heat shock 70 kDa protein 1*β* (HSPA1B) and HSP 90 kDa alpha, class A member 1 (HSP90AA1). Several proteasome subunits (PSMA3, PSMA7, and PSMA8) and cellular structural proteins (Lamin A/C, keratin 18, tubulins, transgelin, etc.) were upregulated by the JIN formula relative to the mock control group. We selected HSP90AA1 for validation by Western blot, which shows that HSP90AA1 was downregulated by the JIN and DPP treatments (Figure 5). The downregulation was more significant in the JIN treatment group compared with that in the DPP group.

## 4. Discussion

Chinese or oriental herbal medicine has long been used for treating cancer. Single herbs are seldom used alone compared with herbal formulae, which uses the synergy and interactions among various phytochemicals present in different herbs to achieve therapeutic efficacy and targets multiple biological and pathological processes while minimizing side effects. 

TCM formulae are rich in potential cancer chemopreventive and therapeutic agents. However, rigorous and systematic evaluations are necessary to establish the efficacy of herbal formulae and transform traditional herbal practices into evidence-based medicine. We evaluated the anticancer activities of the JIN formula, which is an ancient herbal formula recorded in the classic TCM book *Jin Kui Yao Lue* (Golden Chamber). The results showed that the JIN formula significantly delayed the growth of subcutaneous human H460 xenografted tumors in vivo relative to the mock control group. 

Gene array analysis of signal transduction in cancer showed that the JIN formula acted on multiple targets in the MAPK, hedgehog, and Wnt signaling pathways in the H460 xenografted tumor. JIN upregulated two tumor suppressors, namely, CDKN1C and interferon regulatory factor-1 (IRF-1). Abnormal cell cycle regulation is the important reason of excessive cell proliferation and tumorigenesis. Cell cycle progression is regulated by balanced interactions between cyclins and cyclin-dependent kinases (CDKs). The suppressive effect of cyclin-dependent kinase inhibitors (CDKIs) on cyclin/CDK complexes is among the many mechanisms that control normal cell cycle progression. Cell cycle progression is negatively regulated by proteins from two families, the inhibitors of cyclin-dependent kinase 4 (INK4) family [CDKN2A (p16), CDKN2B (p15), CDKN2C (p18), and CDKN2D (p19)] and the CIP/KIP family [CDKN1A (p21), CDKN1B (p27), and CDKN1C (p57)]. The protein encoded by CDKN1C is a tight-binding, strong inhibitor of several G1 cyclin/Cdk complexes and a negative regulator of cell proliferation. Mutations in this gene are implicated in sporadic cancers and the Beckwith-Wiedemann syndrome, thus suggesting that this gene is a tumor suppressor candidate [8]. The anticancer mechanisms of the JIN formula may seek a new breakthrough by the further study of this protein family. IRF-1 was originally identified as a regulator of IFN*α*/*β*. IRF-1 expression is considerably upregulated during viral infections and stimulations by the interferon family. Increasing evidence supports the theory that IRF-1 functions as a tumor suppressor and represses the transformed phenotype. In human tumors, IRF-1 is deactivated to prevent apoptosis and cell cycle arrest by genetic mechanisms [9]. 

The downregulated genes *PTCH2*, *TCF7*, and *WSB1* are related to the embryonic signaling pathways Hedgehog and Wnt. The inappropriate reactivation of these pathways in adult cells promotes tumor growth [10]. Many studies showed that lung tumors are caused by the activation of these embryonic regulatory pathways [11, 12]. Hedgehog signaling plays a key role in a variety of processes, such as embryogenesis, maintenance of adult tissue homeostasis, tissue repair during chronic persistent inflammation, and carcinogenesis. Hedgehog signals protect cancer cells, particularly cancer stem cells [13]. Hedgehog signaling is frequently activated in esophageal cancer, gastric cancer, and pancreatic cancer due to transcriptional upregulation of Hedgehog ligands and epigenetic silencing of HHIP1/HHIP gene, encoding the Hedgehog inhibitor. However, Hedgehog signaling is rarely activated in lung cancer due to negative regulation by the canonical WNT signaling pathway. The Wnt pathway may serve as a potential target in the development of therapeutic agents. The blockade of the Wnt pathway may be considered in formulating new treatment strategies in lung cancer [14]. Given the current and increasing availability of drugs that inhibit Hh and Wnt signaling, an understanding of the role of Hh and Wnt in lung cancer pathogenesis might lead to the development of new therapies. Furthermore, the co-formula treatment of JIN and DDP also targeted the stress/heat shock pathway, specifically for the *HSPA4*, *HSF1*, *MYC*, and *FOS* genes.

HSPs are encoded by several gene families and have essential roles in cell survival, tumorigenesis, and tumor progression. The HSP70 family proteins, which are named according to their approximate relative molecular mass, contain at least eight members that are almost ubiquitously expressed [15]. More than 99% of the amino acids of the two major HSP70 proteins, namely, HSP70-1a and HSP70-1b (encoded by the *HSPA1A* and *HSPA1B* genes, resp.), have been identified. These proteins are initially found in cells under stress [16, 17]. HSP70 is closely involved in programmed cell death protection through interactions with several key regulatory proteins, along with HSP90 and several cochaperones. HSP70-1 proteins are overexpressed in many types of tumor, and this over-expression is often correlated with tumor malignancy, progression, poor prognosis, and metastasis [16]. The overexpression of HSP70-1 induces cell transformation [15]. However, the expression and regulation of HSP70-1 in lung cancer cells are rarely studied. Our analysis showed that HSPA1B, which also belongs to the HSP70 family, was highly expressed in the mock group. After JIN formula treatment, the expression of HSPA1B was downregulated significantly. Cancer metastasis was not observed in the lungs, liver, and brain of mice from the JIN group, thus suggesting that distant tumor metastases was prevented by the JIN formula.

HSP 90 kDa (HSP90) is a molecular chaperone that maintains the function of numerous intracellular signaling nodes utilized by cancer cells for proliferation and survival. HSP90 is also involved in a number of human pathological states such as ischemia and autoimmune diseases. Lung cancer progression is also influenced by HSP90. HSP90*α* is a cytoplasmic protein that is highly conserved in the process of biological evolution. The HSP90 chaperones in humans are encoded by two distinct genes, namely, HSP90*α* and HSP90*β*. Differences in their respective modes of regulation have been observed; HSP90*α* is more inducible than HSP90*β*. HSP90*α* is required for the conformational maturation and stability of multiple oncogenic kinases that induce signal transduction and proliferation of lung cancer cells. HSP90*α* is also important in modulating tumor cell apoptosis. Moreover, HSP90*α* facilitates the migration and proliferation of tumor cells and is associated with the poor prognosis of specific cancers. Changes in the proteins encoded by HSP90 may be caused by changes in the nature and expression of HSP90*α*. These changes may participate in the development of lung cancer. Considering the increased role of HSP90*α* in tumor cells, the expression of the antiapoptotic response of HSP90 in lung cancer incidence and the process of encoding HSP90*α* and HSP90AA1 in single nucleotide polymorphisms may be interrelated and associated with lung cancer. HSP90 is an adenosine trisphosphate- (ATP-) dependent molecular chaperone that maintains the active conformation of clients in coproteins of cancer cells. The inhibition of HSP90 leads to the inhibition of tumor growth and metastasis [17]. HSP90 is also detected on the plasma membrane of tumor cells and its expression is correlated with metastatic potential [18]. In the current study, we discovered that HSP90AA1 was highly expressed in the mock group. This study is the first to report the downregulation of HSP90AA1 by the JIN formula relative to the mock group. HSP90AA1 might serve as a potential prognostic marker or a candidate therapeutic target of H460. HSP90AA1 might also be a viable marker for H460 without distant metastasis.

**Figure 6:**
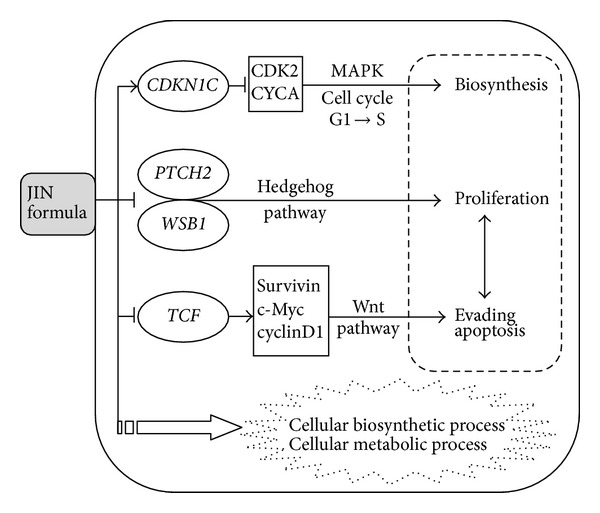
Possible cell signaling network regulated by JIN formula on H460-xonografted tumor.

We found that GO:0044237 (cellular metabolic process) and GO:0006139 (nucleobase, nucleoside, nucleotide, and nucleic acid metabolic process) (Table 5) were enriched by differentially expressed proteins in the JIN group compared with the mock group. HSP90AA1 and HSPA1B were downregulated by the JIN formula. Aldolase A (fructose-bisphosphate) was upregulated in the JIN group compared with that in the mock group. HSP90AA1 is related to GO:0034621 (cellular macromolecular complex subunit organization), GO:0072395 (signal transduction in cell cycle checkpoint), and GO:0044249 (cellular biosynthetic process, etc.). HSP90 is an active ATP-dependent chaperone involved in the assembly and regulation of signal transduction pathways by activating specific client proteins [19]. HSP90AA1 plays an important role in each step of the cell cycle and tumor formation. The function of HSP90 is reflected by the polo-like kinase stability. The inhibition of HSP90AA1 in HeLa cells results in cell cycle arrest either at the G2 stage or metaphase-anaphase transition [20]. Our results suggested that the JIN formula acted on the maintenance of the cell cycle and signaling processes. The potential mechanism of the JIN formula is shown in Figure 6. Further investigation should be conducted to reveal the detailed mechanism of the JIN formula.

## Supplementary Material

We identified 40 peptides (corresponding to 36 proteins) and 178 peptides (corresponding to 84 proteins) that are differentially expressed in the comparison between the MOCK and the DDP group, and the comparison between the MOCK and the JIN group. We found that, the expression of HSP90AA1 and HSPA1B are two most significantly reduced proteins after the JIN group treatment compared with the MOCK group. HSPA1B is also significantly reduced in the DDP group compared with the MOCK group.

## Figures and Tables

**Table 1:** Description of the primer sequences.

Gene	Forward and reverse primer sequences	Annealing temperature (°C)	Length of amplified product (bp)
*β*-*actin *	F: 5′CCTGTACGCCAACACAGTGC3′	59	211
	R: 5′ATACTCCTGCTTGCTGATCC3′		
*CDKN1C*	F: 5′CTGCGGTGAGCCAATTTAGAG3′	59	231
	R: 5′CCTTGGGACCAGTGTACCTTCT3′		
*PTCH2*	F:5′GGCTTCGTGCTTACTTCCA3′	59	259
	R: 5′TGGCGTGCGGTCTGTAT3′		

**Table 2:** Genes regulated more than 2 folds by JIN treatment in H460 cells.

GeneBank	Symbol	Description	JIN/MOCK	*P* value
NM_000076	*CDKN1C*	Cyclin-dependent kinase inhibitor 1C (p57, Kip2)	2.72	0.01
NM_003738	*PTCH2*	Patched homolog 2 (Drosophila)	0.45	0.04
NM_003202	*TCF7*	Transcription factor 7 (T-cell specific, HMG-box)	0.39	0.02
NM_015626	*WSB1*	WD repeat and SOCS box-containing 1	0.13	8.71044*E* − 05

**Table 3:** Genes regulated more than 2 folds by DDP treatment in H460 cells.

GeneBank	Symbol	Description	DDP/MOCK	*P* value
NM_002423	MMP7	Matrix metallopeptidase7 (matrilysin, uterine)	3.16	0.003
NM_001429	EP300	E1A binding protein p300	0.35	0.008
NM_022475	HHIP	Hedgehog interacting protein	0.26	0.02
NM_015626	WSB1	WD repeat and SOCS box-containing 1	0.13	8.79479*E* − 05

**Table 4:** Genes regulated more than 2 folds by JIN + DDP treatment in H460 cells.

GeneBank	Symbol	Description	JIN + DDP/MOCK	*P* value
NM_000076	CDKN1C	Cyclin-dependent kinase inhibitor 1C (p57, Kip2)	3.24	0.003
NM_001904	CTNNB1	Catenin (cadherin-associated protein), beta 1, 88 kDa	0.26	0.003
NM_005228	EGFR	Epidermal growth factor receptor (erythroblastic leukemia viral (v-erb-b) oncogene homolog, avian)	0.46	0.05
NM_001429	EP300	E1A binding protein p300	0.35	0.007
NM_005252	FOS	V-fos FBJ murine osteosarcoma viral oncogene homolog	0.46	0.03
NM_002133	HMOX1	Hemeoxygenase (decycling) 1	0.42	0.09
NM_005526	HSF1	Heat shock transcription factor 1	0.44	0.02
NM_002467	MYC	V-myc myelocytomatosis viral oncogene homolog (avian)	0.22	0.0008
NM_003738	PTCH2	Patched homolog 2 (Drosophila)	0.40	0.01
NM_003202	TCF7	Transcription factor 7 (T-cell specific, HMG-box)	0.32	0.006
NM_015626	WSB1	WD repeat and SOCS box-containing 1	0.13	8.74349*E* − 05

**Table 5:** Significantly enriched GO Biological process terms in the differentially expressed proteins between the JIN formula and the MOCK groups.

GO category	Description	Total genes	Changed genes	Enrichment	log_10_⁡(*P*)
GO:0044237	Cellular metabolic process	4513	45	1.40	−3.26
GO:0044249	Cellular biosynthetic process	2322	26	1.58	−2.22
GO:0006139	Nucleobase nucleoside nucleotide and nucleic acid metabolic process	2350	26	1.56	−2.14
GO:0016032	Viral reproduction	343	16	6.56	−8.93
GO:0034621	Cellular macromolecular complex subunit organization	364	14	5.41	−6.76
GO:0006915	Apoptosis	784	13	2.33	−2.53
GO:0006414	Translational elongation	97	12	17.41	−11.62
GO:0006977	DNA damage response signal transduction by p53 class mediator resulting in cell cycle arrest	57	4	9.88	−3.16
GO:0072395	Signal transduction involved in cell cycle check point	57	4	9.88	−3.16

**Table 6:** Significantly enriched GO biological process terms in the differentially expressed proteins between the DPP and the MOCK groups.

GO category	Description	Total genes	Changed genes	Enrichment	log⁡_10_⁡(*P*)
GO:0006414	Translational elongation	97	3	11.54	−2.68
GO:0065004	Protein-DNA complex assembly	44	2	16.96	−2.22
GO:0071824	Protein-DNA complex subunit organization	51	2	14.64	−2.09
